# Assessment of the Potential of Using Nanofiltration Polymeric and Ceramic Membranes to Treat Refinery Spent Caustic Effluents

**DOI:** 10.3390/membranes12010098

**Published:** 2022-01-17

**Authors:** Ana Isabel Rita, Ana Rita Nabais, Luisa A. Neves, Rosa Huertas, Maria Santos, Luis M. Madeira, Sandra Sanches

**Affiliations:** 1 Sines Refinery, Petrogal S.A., 7520-952 Sines, Portugal; aibr1910@gmail.com (A.I.R.); maria.santos@galp.com (M.S.); 2 LEPABE-Laboratory for Process Engineering, Environment, Biotechnology and Energy, Faculty of Engineering, University of Porto, Rua Dr. Roberto Frias, 4200-465 Porto, Portugal; mmadeira@fe.up.pt; 3 LAQV/REQUIMTE, Chemistry Department, Nova School of Science and Technology, Universidade NOVA de Lisboa, 2829-516 Caparica, Portugal; a.nabais@campus.fct.unl.pt (A.R.N.); lan11892@fct.unl.pt (L.A.N.); rosa.huertas@ibet.pt (R.H.); 4 IBET-Instituto de Biologia Experimental e Tecnológica, Apartado 12, 2780-157 Oeiras, Portugal

**Keywords:** naphthenic spent caustic effluent, nanofiltration, aging, ceramic vs. polymeric membranes

## Abstract

Spent caustic effluents are very challenging due to their very hazardous nature in terms of toxicity as well as their extreme pH (approximately 12–14). Spent caustic has presented a challenge for wastewater treatment in refineries, due to its composition rich in mercaptans, sulfides and other aromatic compounds. To address such problems, membrane filtration was studied using real effluents from Sines Refinery, in Portugal. The present study attempts to assess the potential for spent caustic treatment with nanofiltration (NF) polymeric and ceramic membranes, assessing membrane life expectancy. For that, membrane aging studies in static mode were performed with the polymeric membrane before attempting NF treatment (dynamic studies). A ceramic membrane was also tested for the first time with this type of effluents, though only in dynamic mode. Although the polymeric membrane performance was very good and in accordance with previous studies, its lifespan was very reduced after 6 weeks of contact with spent caustic, compromising its use in an industrial unit. Contrarily to expectations, the ceramic membrane tested was not chemically more resistant than the polymeric one upon direct contact with spent caustic (loss of retention capacity in less than 1 h in contact with the spent caustic). The results obtained suggest that a pH of 13.9 is very aggressive, even for ceramic membranes.

## 1. Introduction

Worldwide, attempts have been made to treat complex wastewaters and assess the separation of recalcitrant organic compounds by membrane filtration. Wastewater compositions may vary completely, some of them being toxic to human beings and the environment. Most of these wastewaters are produced by heavy chemical industries, such as metallurgy, refineries and the paper industry.

In the specific case of refinery wastewaters, the treatment of spent caustic is presently a challenge due to its composition rich in mercaptans, sulfides, aromatic structures with ketones and alcohols, etc. [1]. The mercaptans that are extracted to the caustic solution in the kerosene prewashing step, along with naphthenic acids and other organosulfur species, constitute naphthenic spent caustic [2,3,4,5,6,7,8]. Due to the very high pH value of spent caustic, around 13, this wastewater becomes extremely difficult to handle [9,10].

Easy scale-up, low-temperature operation and space-efficiency of plants are among some of the advantages of the use of membranes for fluid filtration. However, fouling of the membrane and even complete destruction of its structure are common problems that make membrane selection critical [11]. Aging studies have been performed to understand membrane degradation and loss of performance with time when exposed to a certain agent under study [11,12,13,14,15,16,17,18,19,20,21]. Such studies usually assess membrane resistance to cleaning procedures such as oxidant agents (e.g., hypochlorite) or alkaline agents (e.g., NaOH). Most of the aging studies assess the impact of cleaning agents on membranes to evaluate the membrane integrity over time due to cleanings carried out during short periods of time with a certain frequency. In this context, several characterization techniques are usually applied to address membrane modifications (active layer, cross-section and support layer), such as scanning electron spectroscopy (SEM), energy dispersive spectroscopy (EDS) and Fourier-transform infrared spectroscopy (FT−IR) [22].

Nanofiltration (NF) has been applied to treat complex wastewaters with recalcitrant compounds [23,24,25,26]. Several membrane filtration processes have been studied to treat refinery effluents, although the studied effluents usually present milder pH values and organic compound concentrations compared to spent caustic; for example, Salahi et al. [27], Abadi et al. [28], Ishak et al. [29], Shariati et al. [30], Zhong et al. [31] and Zhu et al. [32] only refer to oily wastewaters, which usually mean end-chain wastewater with free and/or emulsified oil particles.

Polymeric membranes are the most common membranes used for NF treatment; however, ceramic membranes have also started to be considered as their molecular weight cut-off (MWCO) has been decreased, alongside their greater chemical stability. A study on chemical cleaning of an Inopor ceramic membrane, similar to the one tested in the present study, showed that the alkaline agent NaOH seems to compromise membrane performance significantly [33]. Not many studies exist for low MWCO ceramic membranes addressing membrane performance during aging studies [34] and/or membrane separation processes such as NF, especially for extreme pH effluents, like spent caustic.

Studies with other matrixes show that, for example in the removal of polycyclic aromatic hydrocarbons (PAHs) from wastewaters, size exclusion may be the primary removal mechanism for ceramic membranes while, for example, hydrophobic interactions played a minor role [35]. Zhao et al. [35] studied the impact of organic fouling on ceramic and polymeric membranes, concluding that ceramic membranes present a higher resistance to fouling and that their permeability can be restored after chemical cleaning cycles. Given the possible propensity for fouling formation in complex matrixes with a higher concentration of organics, it seems that ceramic membranes could enable an effective separation. A Koch MPF-34 polymeric membrane and a TiO_2_ ceramic membrane supported on Al_2_O_3_ were used to treat acid-mine waters (pH 1.0) with positive results (maximum concentration factor of 4.19 for the polymeric membrane and 3.29 for the ceramic membrane to achieve 80% of permeate recovery, which was the maximum recovery tested). However, the authors refer to a need for a lower MWCO ceramic membrane [36]. From the work of López et al. [36], it seems that both ceramic and polymeric membranes tested may be chemically resistant to very low pH values, but no conclusion is made regarding very high pH values. A study performed by Dalwani et al. [37] addressed NF performance with a modified polyethersulfone (PES) composite membrane in acidic and alkaline media, observing a stable membrane performance even after prolonged exposure (up to several weeks). The authors reported that especially in strongly alkaline conditions, the membrane appeared to have wider pores; they describe the phenomenon as membrane matrix swelling caused by different interactions between the ions in the solution and the resulting membrane charge density at different pH. Santos et al. [24] attempted to treat refinery spent caustic with an NF Koch MPF-34 spiral wound configuration polymeric membrane. In their study, very high rejections were obtained for key components of spent caustic effluents (99.9% for oil and grease and 97.7% for chemical oxygen demand (COD)) at the optimal concentration factor of 3. On the other hand, this study did not include testing the membrane in the long term (i.e., assessing retention and permeability profiles with time) or assessing the main structure modifications due to high pH exposure time, making these results only valid for first-time application (fresh membrane). Therefore, more studies are necessary about the lifespan of this membrane (and others) with refinery spent caustic and will provide feedback for a potential industrial treatment.

The present study attempts to assess the potential for spent caustic treatment with NF polymeric and ceramic membranes. For that, membrane aging studies in static mode were performed with the polymeric membrane before testing its performance for spent caustic treatment (under dynamic conditions), and results were compared with the work performed by Santos et al. [24]. A ceramic membrane was also tested, though only in dynamic conditions. To the best of the authors’ knowledge, previous studies did not evaluate membrane aging in filtration mode throughout long-term operations, also with the particularity of such an aggressive effluent.

## 2. Materials and Methods

### 2.1. Reagents

H_2_SO_4_, 95–97%, was provided by Fluka, Honeywell (Morristown, NJ, USA); NaOH pellets, reagent grade, were supplied by PanReac; silica gel (grade 923) was provided by Grace Davidson (Columbia, MD, USA); tetrachloroethylene was provided by PanReac AppliChem (Barcelona, Spain); and NaHSO_3_ was provided by AnalaR NORMAPUR, VWR (Radnor, PA, USA).

Cotton filter papers, grade 40, with a diameter of 150 mm and a pore size of 8 µm were provided by Whatman, GE (Little Chalfont, Buckinghamshire, UK). This material was used as part of SMEWW5520 C/F—analysis of oil and grease in wastewater (cf. Section 2.6.1).

### 2.2. Membranes

Both polymeric and ceramic nanofiltration membranes were assessed in terms of their potential towards naphthenic spent caustic effluents. The commercial proprietary composite polymeric Koch membrane, flat-sheet type (18′ × 18′) SeIRO MPF-34 with a MWCO of 200 Da, was selected based on its stability under acid/base conditions as well as promising results previously obtained [24]. A commercial ceramic NF membrane from Inopor (Germany) with active and support layers composed of titania (TiO_2_) and alumina (Al_2_O_3_), respectively, was also studied. This is a single-channel tubular membrane with a MWCO of 200 Da and a filtration area of 0.011 m^2^.

### 2.3. Experimental Setup

**Figure 1:**
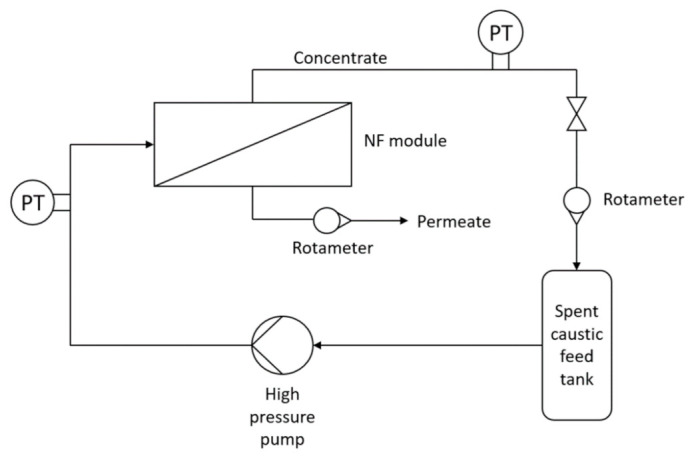
Schematic representation of the NF experimental setup.

The assessment of NF membranes’ potential to treat spent caustic effluents was conducted in a bench-scale unit, illustrated in Figure 1.

The bench-scale unit consisted of two vessels for the feed and the permeate, an Ifimoto Iberica centrifugal high-pressure pump and a manual valve, through which the pressure inside the system could be adjusted. Transmembrane pressure (TMP) was measured with two pressure transducers from Aplisens (model WW-45) placed before and after the NF module. Pretreatment of the effluent was ensured with an 80 µm prefilter, which can retain solid particles that could promote membrane fouling.

The bench-scale unit in this setup is very versatile, as it allows testing different types of membrane configurations and even MWCO ranges. The NF module in Figure 1 may represent a crossflow module with the polymeric flat sheet membrane or a tubular module with the ceramic tubular membrane. For the crossflow module, a stainless steel crossflow cell was used with a 15 cm^2^ flat-sheet SeIRO MPF-34 membrane (Koch, Spain) that was placed between two rectangular cross-section channels (feed and permeate) measuring 150 mm × 10 mm × 1 mm (length × wide × height) each. In the case of the tubular configuration, a stainless steel tubular single-channel housing of 500 mm × 10 mm (length × internal diameter) was used with a 110 cm^2^ tubular single-channel ceramic membrane, a TiO_2_ with 200 Da (Inopor, Germany).

### 2.4. Aging Experiments

Membrane aging is typically employed to address membrane degradation, where alterations can be evaluated in both the active layer and the support. Aging experiments were executed firstly in static conditions for 12 weeks in duplicate assays and secondly in dynamic conditions for 6 and 12 weeks (static aging prior to NF test).

The aging experiments in static conditions allowed studying possible alterations in membrane dimensions and contact angle values and were performed in duplicate with 4 cm^2^ membrane (2 × 2 cm^2^). The 4 cm^2^ pieces were individually soaked in the aging solution (spent caustic effluent No. 1 (see Table 1)) inside glass flasks and were kept in the dark and under static conditions for 12 weeks. Dimensions were taken each two weeks, while the contact angle measurements were performed at the end of the 12 weeks. The contact angles of the polymeric membranes were measured to evaluate their hydrophilic/hydrophobic nature over the soaking time while the dimensions were taken in order to evaluate a possible loss of volume (cf. Section 2.6.2). To compare with the pristine membrane, another 4 cm^2^ piece was rinsed with distilled (DI) water before analyses.

The aging experiments in dynamic conditions were performed with two pieces of polymeric membranes of 15 cm^2^ each that were cut to enable a perfect fitting in the filtration unit for subsequent filtration tests. These membranes were rinsed with DI water to remove all preservatives and then characterized as described in Section 2.6.2. After that, the pieces were individually soaked in the aging solution (spent caustic effluent No. 1 (Table 1)) inside glass flasks and were kept in the dark and under static conditions for 6 or 12 weeks. After these periods, the aged membranes were taken, rinsed with DI water and placed in a NaHSO_3_ solution (5 g/L) for preservation until nanofiltration tests were carried out. Then, the aged membranes and the pristine membrane (used as control, rinsed with DI water before NF test) were submitted to NF tests (described in Section 2.5), after which were characterized by means of scanning electron microscopy–energy-dispersive X-ray spectroscopy (SEM-EDS) and Fourier-transform infrared spectroscopy (FT−IR), in order to draw conclusions about possible structure modifications caused by changes in the retention capacity and permeability over time, as a means to simulate long-term industrial treatment processes.

### 2.5. Nanofiltration Experiments

The hydraulic permeability for both polymeric and ceramic membranes was determined prior to any experiment. Polymeric membranes were cut in rectangles of 15 cm^2^ of filtration area and placed in the crossflow module. The ceramic membrane was directly placed in the Inopor housing. Water permeability was determined by filtering DI water at 10.0 bar, 8.0 bar and 6.0 bar for 2 h (also ensuring compaction of the polymeric membrane for NF tests). Permeate weight was measured over time using a 250 mL beaker. Temperature throughout the test was monitored with a thermometer (VWR). Temperature and transmembrane pressure (TMP) were measured every 15 min during water permeability tests. Permeability, *L_P_*, was calculated as shown in Equation (1), where *V_P_* stands for permeate volume, *t* stands for the duration of a permeate sample collection, *A* stands for filtration area and Δ*P* stands for the TMP. No correction was included for temperature.

(1) $$L_{P}=\frac{V_{P}}{t\times A\times \Delta P}$$

Another important parameter in assessing membrane performance is compound rejection. The apparent rejection (R) can be calculated as shown in Equation (2), where *C_P_* and *C_F_* are the concentrations of a given compound in the permeate and feed, respectively.

(2) $$R\;\left(\%\right)=100\times \left(1-\frac{C_{P}}{C_{F}}\right)$$

After hydraulic permeabilities were determined, the membrane performance was assessed through NF tests. Nanofiltration experiments with polymeric membranes were performed with effluent No. 1 (Table 1) and conducted at 10 bar with a cross-flow velocity of 1.22 m/s (equivalent to a flow rate of 2.82 L/min), representing a Reynolds number (Re) of 2485 (almost laminar flow). Nanofiltration experiments with ceramic membranes were performed with effluent No. 2 (Table 1) and were conducted at 10 bar with a cross-flow velocity of 1.93 m/s (equivalent to 4.45 L/min), representing a Re of 15,134 (which is turbulent).

It was necessary to apply a pretreatment to the raw effluent in order to remove particulates and oily droplets that could cause fouling on the membrane. The pretreatment was also carried out in the experimental setup (Figure 1), where the NF module was replaced by the prefilter. A total volume of 100 L of naphthenic spent caustic was prefiltered before the NF tests were executed. NF tests were carried out in concentration mode, meaning that the permeate was collected in a different flask and the retentate was recirculated to the feed flask. Because the flux was so low during NF tests, it was not possible to perform flux intermediate measurements during each test. Therefore, each test lasted until enough permeate volume was collected to perform all the physicochemical analyses (around 7 h for pristine polymeric membrane tests, and approximately 1.3 h for each aged membrane). After the conclusion of the NF test, a sequence of basic and acidic washings was applied. The NF unit was rinsed first with tap water to remove the working solution, and then the unit was rinsed with DI water. After that, the basic cleaning focused on feeding the system with a sodium hydroxide solution (5 g/L) recirculated for 15 min. After rinsing the system with DI water, an acid cleaning with a citric acid solution (5 g/L) was carried out for 15 min, followed by a final rinsing with DI water. Hydraulic permeabilities were taken in each step. At the end of the cleaning procedure, the unit was fed with a solution of sodium bisulfite (5 g/L) to preserve the membrane for future use.

In the case of the pristine polymeric membrane, two more NF tests were attempted to test the robustness of the membrane, modifying the pH of the spent caustic stream feeding the NF unit. Spent caustic (effluent No. 1 in Table 1) pH was lowered to 10.0 and to 3.0 with H_2_SO_4_. In the case of pH 3.0, two phases were formed; the phase of interest was the polar one, so only this phase was collected to be treated by NF. In the case of ceramic membranes, the 200 Da membrane was tested only with the spent caustic at its original pH (effluent No. 2 in Table 1).

All samples throughout the study were characterized, as described in the following section, in terms of concentrations of contaminants such as COD, oil and grease (O&G) (polar and nonpolar), phenolic compounds and sulfides; pH; and conductivity in order to evaluate the retention capacity of the membranes. Before and after NF tests, the polymeric membrane was characterized by SEM-EDS and FT−IR, and the ceramic membrane was characterized by SEM-EDS (as described in Section 2.6.2). Inductively coupled plasma atomic emission spectrometry (ICP-AES) was used to determine the concentration of metal elements, namely titanium and aluminum, in spent caustic solutions (feed, permeates collected during the test, total permeate and retentate) in the NF test carried out with the 200 Da ceramic membrane (see Section 2.6.2).

### 2.6. Characterization Methods

#### 2.6.1. Effluent Characterization

COD was measured using the LCK 014 kit (HACH) and a DR 3900 spectrophotometer (HACH) after the digestion of the samples in an HT 200 S digester (HACH LANGE), according to APHA guide [38]. Sulfides and phenolic compounds were determined by spectrophotometry using a DR 3900 spectrophotometer (HACH) as well as the LCK 653 kit and the LCK 346 kit from HACH, respectively. O&G (polar and nonpolar) was measured according to the Standard Method SMEWW 5520 C/F [38], after sample dilution to ensure that the concentration would fit into the calibration curve of the NICOLET 6700 FT−IR instrument. The conductivity was measured using a conductivity meter, model LF 320 (WTW); pH was measured using a pH 1100 L pH meter (VWR), and temperature was measured with an alcohol thermometer (VWR).

#### 2.6.2. Membrane Characterization

##### Dimensions and Contact Angle

The thickness of the polymeric membranes was monitored with a micrometer (Elcometer 124, Manchester, UK).

The contact angles were determined by the sessile drop method, by manually depositing a drop of DI water and spent caustic on the membrane surface. Multiple replicates (9 measurements on 3 different locations of the active layer and support) were performed and the mean angle was determined. All images were acquired by CAM2008 (KSV) software, where the drop shape fitting was executed by mathematical functions.

##### Scanning Electron Microscopy with Energy Dispersive Spectroscopy (SEM-EDS) and Mapping

Scanning electron microscopy (SEM) with energy dispersive spectroscopy (EDS) analyses were performed on polymeric membranes, while SEM with element mapping was performed on the surface of ceramic membranes. The morphology of the support and active layers of the membranes was studied by a Hitachi S-2400 scanning electron microscope. The microscope was equipped with a silicon drift detector (SDD) to characterize the elemental composition of the surface based on the EDS spectroscopy and a digital acquisition system with Esprit 7.1 software to detect Bruker light elements. Prior to SEM-EDS and SEM mapping characterizations, membranes were coated with gold to make them electrically conductive.

Cross-sections of each membrane were processed using the software ImageJ (University of Wisconsin, Madison, WI, USA) [39,40] for the ×300 SEM image amplification obtained. The images were spatially scaled, and their total membrane surface area was calculated and then binarized after setting a certain threshold level (149 for pristine polymeric membrane and 255 for aged polymeric membranes). Pore density (number of pores divided by area of membrane), porosity (area of pores divided by membrane area), total pore area and the average Feret’s diameter (the longest distance between any two points along the selection boundary) were further automatically determined by the software.

###### Fourier-Transform Infrared Spectroscopy (FT−IR)

FT−IR coupled with attenuated total reflectance (ATR) was used to analyze and compare the surface chemical structure of membranes on both sides (active and support layers). Bruker Spectrometer IFS 66/S instrument (USA) equipped with an H-ATR and a ZnSe crystal was used as equipment, and normalized spectra were recorded with accumulated 60 scans in the range of 4000 to 550 cm^−1^ and with 4 cm^−1^ resolution. All membranes were cleaned with DI water and dried before analysis. Three different random zones were selected for the analysis of each membrane layer to assess potential differences in the chemical structure in different membrane sections.

##### Inductively Coupled Plasma Atomic Emission Spectroscopy (ICP-AES)

The equipment used for ICP-AES analyses was a Horiba Jobin Yvon S.A.S., Model Ultima2 (France). This was equipped with a 40.68 Hz RF generator and a Czerny-Turner 1.00 m spectrophotometer. Data acquisition and equipment control were performed with a computer equipped with JY v5.4 software, which allows visualizing all parameters and obtained data online. Sample volume was 1 mL per metallic element.

## 3. Results and Discussion

### 3.1. Effluents

Spent caustic is the solution that results from the kerosene prewashing step, before the sweetening process, in the kerosene merox unit of a petroleum refinery. In the specific case of Sines Refinery, in Portugal, this extremely aggressive effluent accounts for 90% of the impact on the final refinery wastewater in terms of polar oil and grease (O&G) [41].

The characteristics of spent caustic vary over time, due to the different crudemix being processed in the refinery and the operating conditions employed. Two different spent caustic effluents were tested due to constraints related to the plant operation procedures: one to assess NF on polymeric membranes (effluent No. 1) and the second collection to assess NF on ceramic membranes (effluent No. 2). Moreover, the robustness of the polymeric membrane was tested at different feed pH. Table 1 exhibits the compositions of both effluents.

### 3.2. Koch SeIRO Polymeric Membrane

#### 3.2.1. NF Tests to Evaluate Membrane Performance

Pure water permeability is an intrinsic membrane characteristic mainly governed by the active layer pore size distribution. The hydraulic permeability for the polymeric membrane was determined to be 1.81 ± 0.28 Lh^−1^bar^−1^m^−2^, at 22 ± 2 °C. The fabricant of the polymeric membrane, Koch, lists a hydraulic permeability from 1.5 to 2.0 Lh^−1^bar^−1^m^−2^, which is in line with the obtained experimental value.

Membrane performance was addressed after aging for 6 and 12 weeks with spent caustic (effluent No. 1 in Table 1). The values of *L_P_*/*L_P_*_0_ (hydraulic permeability at a given time normalized by the hydraulic permeability of the pristine membrane) obtained for 6 weeks and 12 weeks aged membranes were, respectively, 4.46 and 4.62. A considerable increase in the hydraulic permeability of the membrane after soaking in the spent caustic effluent can be observed. It is interesting that the increase in the permeability mainly occurs within the initial period of 6 weeks since similar permeabilities were attained after 6 and 12 weeks of aging (8.07 ± 0.31 Lh^−1^bar^−1^m^−2^ vs. 8.36 ± 0.34 Lh^−1^bar^−1^m^−2^ after 6 and 12 weeks, respectively).

**Figure 2:**
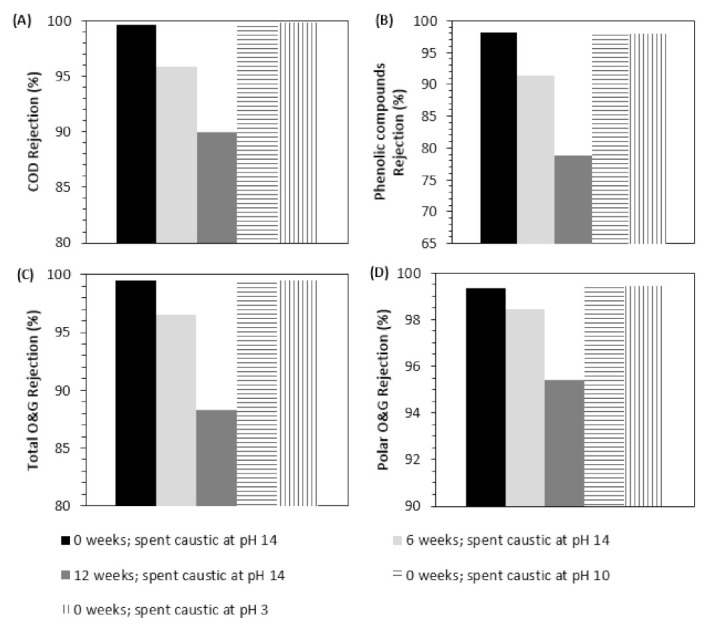
Apparent rejection of contaminants during membrane separation with aged membranes for 0 (pristine membrane), 6 and 12 weeks with spent caustic at pH 14 and pristine membranes with spent caustic at pH 10 and 3. (**A**) COD, (**B**) phenolic compounds, (**C**) total O&G, (**D**) polar O&G.

Figure 2 shows the rejection of contaminants during membrane separation tests. COD removals were very high for the pristine membrane (99.6%); however, a continuous decrease is observed with the aging time (95.8% for 6 weeks and 90.0% for 12 weeks aged membranes (Figure 2A)).

Considering the first 6 weeks of aging, COD removal decreased on average 0.63% per week, but in the second period of 6 weeks, COD removal decreased on average 0.97% per week. For the membranes aged for 6 and 12 weeks, phenolic compound removals reached 91.3% and 78.9%, respectively, which are much lower than the pristine membrane performance (98.1%) (Figure 2B). It should be noted that phenolic compounds are the target compound class with the lowest MW of all (180–220 Da) [42]; therefore, it may be deduced that the more significant loss of retention is for phenolic compounds. The decrease in their rejection is aligned with the decrease in membrane retentive properties and increase in membrane permeability, which may be related to swelling effects [37]. Sulfide compounds are not presented in Figure 2 since their rejection is 100% in all samples, which may be related to the natural degradation of sulfides into the atmosphere (NF tests occurred during summertime in the open air). Total O&G removal reached a maximum of 99.5% for the pristine membrane. Considering aged membranes, the performance in O&G removal decreased also (96.6% removal for 6 weeks aged membrane and 88.3% removal for 12 weeks aged membrane) (Figure 2C). For aged membranes, polar O&G removals reached 98.4% for 6 weeks and 95.4% for 12 weeks (0.15% average removal loss per week during the first 6 weeks of aging and 0.51% average removal loss per week during the second 6 weeks of aging) (Figure 2D). These results indicate a loss of rejection of polar O&G contaminants on aged membranes, especially for the 12 weeks aged membrane. This loss of retention capacity was more relevant during the second 6 weeks of aging.

As mentioned previously, the major increase in permeability occurred during the first 6 weeks of aging, while the highest decreases in the rejection capacities were observed during the second 6 weeks of aging. The small increase in permeability in the second 6 weeks of aging seems not to be able to justify the increase in the loss of retention capacity in the same period. It could be related to molecular exclusion mechanisms, as it seems that the membrane pore integrity may have been compromised during the aging period, as reported elsewhere [11,12,13,15,16,18,19,20,22].

One possible reason to change membrane pore integrity in such a way that compromises permeability and retention capacity could be the very high pH of the spent caustic. Data from FT−IR observations (described hereinbelow) suggest that the active layer is not likely to have been modified since the differences observed are related to the adsorption of organic components that are present in spent caustic. However, permeability is a property largely governed by the narrowest pore (that of the active layer), which leads to the conclusion that, to some extent, permeability data variations observed with aging are related to the possible deterioration of the active layer, although not observable. On the other hand, the support layer seems to have been considerably modified, according to the alterations in retention properties.

Although the membrane is to some extent pH-resistant, it could happen that the extreme pH conditions negatively affect the membrane structure. In order to test this, spent caustic pH was decreased from 14 to 10 and 3. The values of *L_P_*/*L_P0_* obtained for pH of 14, 10 and 3 were, respectively, 3.04, 2.37 and 2.07. Hydraulic permeability seems to suffer more the higher the pH of the solution, particularly between pH 10 and 14. Figure 2 shows the impact of pH on the retention capacity of the polymeric membrane. Retention capacity of the pristine membrane is nearly independent of the pH value, with all contaminant rejections being similar in all experiments. Therefore, it can be concluded that pH manipulation will only cause an alteration in the permeability and is not responsible for retention capacity alterations.

Santos et al. [24] attempted to treat naphthenic spent caustic with a Koch NF polymeric SeIRO spiral-wound membrane module. It was suggested that the contaminants present in the spent caustic do not significantly affect the membrane performance. The results herein obtained corroborate the study of Santos et al. [24] for a pristine membrane; however, a great loss of performance was observed for aged membranes: a great loss of performance in permeability during the first 6 weeks of ageing and a great loss of performance in retention capacity in the second 6 weeks of ageing.

#### 3.2.2. Contact Angle and Thickness

Contact angle depends highly on the surface composition and, to a lesser extent, on the surface roughness. The contact angle is a measure of the tendency for the water to wet the membrane surface (it can be measured on both sides). For the Koch SeIRO polymeric membrane, the contact angle with DI water on the active and support layers was 30.62° and 36.28°, respectively, showing the hydrophilic nature of the membrane on both sides. Interestingly, the contact angle with spent caustic was approximately 0° on both sides, demonstrating the higher affinity of the membrane towards the spent caustic effluent, potentially towards polar organic compounds (with a polarity lower than the water polarity).

**Figure 3:**
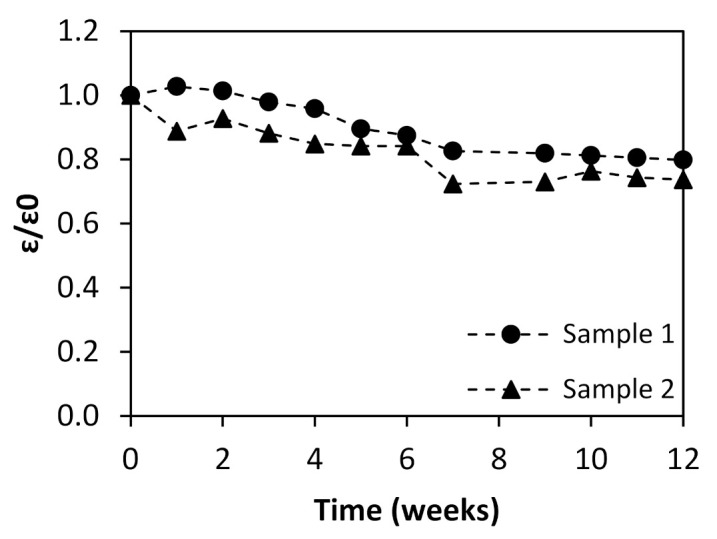
Thickness profile measured for the two immersed polymeric membranes along the 12 weeks of aging (ε—thickness at any time during the aging procedure; ε_0_—thickness at the beginning of the aging procedure).

Figure 3 presents the thickness evolution during 12 weeks of aging in duplicate samples. In the present work, both length and width were determined as 2 cm for all sampling points throughout the 12 weeks.

Figure 3 shows a consistent loss of thickness for the two studied membranes, and therefore a loss of membrane volume is observed during aging. This observation could be related to modifications to the membrane structure on the active layer, support or both. These results are consistent with permeability and retention property data obtained and discussed above, in the previous section.

#### 3.2.3. SEM-EDS

**Figure 4:**
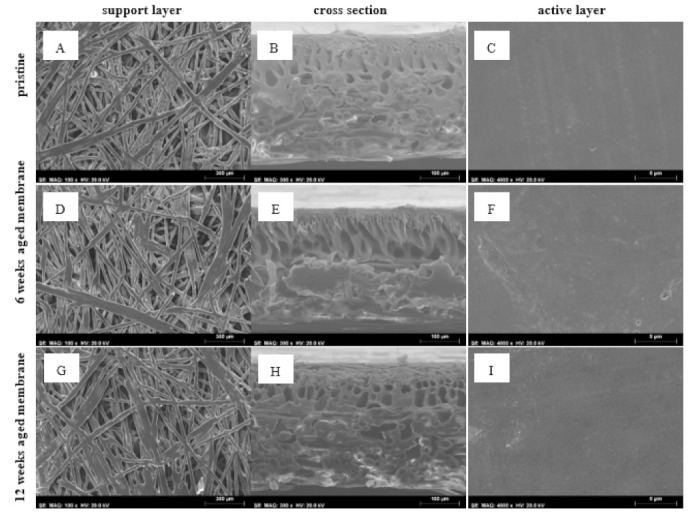
SEM analysis for the polymeric membrane. (**A**) Pristine membrane support layer; (**B**) pristine membrane cross-section; (**C**) pristine membrane active layer; (**D**) 6 weeks aged membrane support layer; (**E**) 6 weeks aged membrane cross-section; (**F**) 6 weeks aged membrane active layer; (**G**) 12 weeks aged membrane support layer; (**H**) 12 weeks aged membrane cross-section; (**I**) 12 weeks aged membrane active layer. Support layer magnification is ×100, cross-section magnification is ×300 and active layer magnification is ×4000.

Active and support layers as well as cross-sections of pristine and aged membranes (for 6 and 12 weeks) were analyzed by scanning electron microscopy (SEM). Figure 4 shows the results obtained.

In terms of physical modifications of the membrane structure, it appears that the intermediate structure (right after the active layer in the cross-section view) becomes slightly looser with aging (Figure 4B,E,H), although it is not completely clear. Pore coalescence may be the cause for this observation. The support layer (Figure 4A,D,G) seems unchanged. No conclusions can be drawn regarding the active layer since the structure is very compact and SEM maximum magnification does not enable comparisons among the membranes with the required level of detail. On the other hand, variations in permeability and thickness were observed with time, thus making it impossible to draw a clear conclusion of a connection between membrane structure and function (*L_P_*, and rejection capacity) based on SEM results alone. To further study membrane structure, FT−IR profiles were collected and are discussed in the next section. Chemical surface composition (EDS) was also studied to determine elementary composition (Table 2).

It is interesting to observe a continuous increase in the carbon content with aging in the active layer while other elements (S and O) are decreasing, which could mean a possible degradation of this layer. On the support, an alteration occurs between 6 and 12 weeks of aging and is related to a loss of sulfur. Although the membrane composition is not available, SEM-EDS analysis suggests that its composition essentially includes carbon, oxygen and sulfur in the active layer and mostly carbon in the support. Especially for the active layer, which has the selectivity property, several polymers used in NF polymeric membranes contain such composition, such as polyethersulfone (PES) and polysulfone (PS) [43].

Pore density analyses were performed on the pristine membrane, as well as for the 6 and 12 weeks aged membranes, prior to NF tests. An SEM cross-section image (×300 image magnification) of each membrane was processed using the ImageJ software. For that, the total cross-section area was calculated over SEM images that were also binarized (Figure S1 in the Supplementary Material section), and the values obtained are summarized in Table 3, comprising only the pore features in the membrane support (due to the very low MWCO of the active layer, SEM could not show any pores). As such, the discussion of the impact of membrane aging may only be performed considering the main porous parameters for the membrane cross-section. However, a cross-sectional analysis also has some limitations since it only indicates possible distortion of the pores due to membrane aging.

The cross-section area obtained, calculated pore density (number of pores divided by area of membrane), porosity (area of pores divided by area of membrane), circularity (Equation (3)) and Feret’s diameter (the longest distance between any two points along the selection boundary) [44] are presented in Table 3.

(3) $$\mathrm{Circularity}=4\pi \frac{\mathrm{Area}}{{\mathrm{Perimeter}}^{2}}$$

From the data presented in Table 3, is clear that the pores in the support are of micro scale, even in the pristine membrane. However, the membrane support aged 6 and 12 weeks showed an increase in the porosity, number of pores and pore density. Extremely high Feret’s diameter values were detected, indicating the highly irregular shape of pores, particularly for the 6 weeks aged membrane. The estimated average circularity value was around 0.8 in all measurements with estimated values obtained that varied between 0.6 and 1 (perfect circles) but with a high standard deviation (Figure S2). Therefore, a considerable variation in the average Feret’s diameter values was also obtained, indicating a highly irregular shape of pores in the support layer, particularly finger-like for this part of the membrane support. Moreover, the increase in the maximum Feret’s diameter obtained for the 6 weeks aged membrane indicates a possible distortion of the porous structure (clearly shown for the wider porous section area) after aging. On the other hand, the decrease in this value with the aging time indicates a possible compaction at the end of 12 weeks.

#### 3.2.4. FT−IR

The objective of these studies was to determine the possible structure alterations in the membranes that occurred during aging experiments and after NF tests. The tested membranes are divided as the pristine membrane without any treatment (“Pristine”) and with an NF test (“Pristine + NF test”), the duplicate aged membranes after immersion for 12 weeks (“Membrane 1 + 12 weeks” and “Membrane 2 + 12 weeks”) and the membranes aged for 6 and 12 weeks followed by an NF test (“Membrane 6 weeks + NF test” and “Membrane 12 weeks + NF test”). It is important to mention that both the side in contact directly with the effluent in the NF tests (active layer) and the opposite side (support layer) were tested.

**Figure 5:**
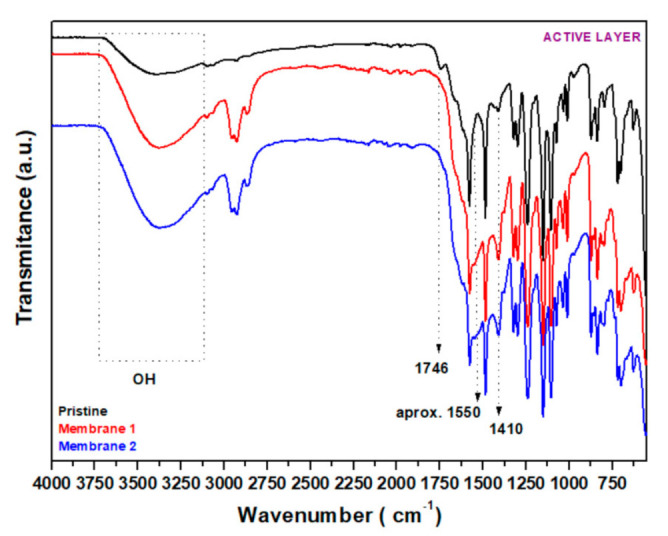
FT−IR spectra of the active layer of the pristine membrane and of the active layer for membranes after 12 weeks immersion in spent caustic (made in duplicate).

The FT−IR spectra obtained for the active layers for two membranes immersed under the same conditions in the effluent were analyzed and compared with the membrane that was not immersed; data are shown in Figure 5.

The results obtained by FT−IR for the active layer of 12 weeks aged membranes after immersion (duplicate: Membrane 1 and Membrane 2) depicted in Figure 5 and amplified in Figure S3 were nearly identical but showed some differences compared to the pristine membrane. Thus, among similarities, bands associated with the C=C bond at around 3100 cm^−1^, 871 cm^−1^ and 833 cm^−1^ (Figure 5 and Figure S3) were identified in all spectra. The pristine and both the 12 weeks aged membranes showed bands at around 3370, ~3100, 2950–2924 and 2860 cm^−1^ (Figure S3A), characteristic of –OH, =C-H, Cl-C-H and H-C-H bonds, respectively [45]. The peak at 1142 cm^−1^ was associated with C-O st and was also present in all spectra, as were peaks in the range of 800–600 cm^−1^, also assigned to C=CH stretching vibration [45] and occasionally C-Cl [46,47]. A peak at around 1576 cm^−1^ was also observed in all spectra, probably associated with the stretching mode of -COO st group. Peaks at 1665 cm^−1^ and 1435 cm^−1^ were linked to asymmetric and symmetric vibration of -COO- groups, respectively [48,49], and peaks at 1070 and 1031 cm^−1^ associated with S=O bonds were also present, seeming to be in accordance with the presence of those atoms in SEM-EDS results (Table 2).

Among the differences observed, the pristine membrane spectrum presented a characteristic band at 1746 cm^−1^ due to the stretching vibration of the C=O group, while for the 12 weeks aged membranes this peak disappeared (Figure 5 and Figure S3B). This fact could be attributed to the aminolysis reaction with the urethane groups with ammonia or alkanolamines, present in the effluent and giving as subproducts alcohols and amides, as previously reported [50]. Moreover, this is in line with the increase in intensity observed in the spectra for the band associated with these groups (OH bond), around 3370 cm^−1^ (Figure 5) and 1410 cm^−1^, as well as the differences in the C=O band in the amide region (around 1690–1630 cm^−1^) (Figure S3B). The increase in the intensity for bands at 2950 cm^−1^ and 2924 cm^−1^ in the spectra of 12 weeks aged membranes could be attributed to the presence of compounds from the effluent (Figure S3A). A broad band appearing at around 1520 cm^−1^ is associated with the presence of ammonia (NH_4_^+^) and salt derivates (-NH_3_^+^) and could be also related to the effluent. Bands at around 1550–1550 cm^−1^ associated with N–O asymmetric stretch; 1377 cm^−1^, symmetric stretch; and 773 cm^−1^, out-of-plane stretch modes for deformation of nitro compounds (nitrates (NO_3_^−^) and nitrites (NO_2_^−^)) [45], which are present as residues in the aged membranes, are also often found in the spectra of other oily effluents [51]. New peaks at 809 cm^−1^ and at 685 cm^−1^ are also associated with the effluent residue.

Thus, after analyzing and comparing the spectra, we can conclude that the immersion of the membranes does not have an impact on the main bands associated with the membrane initial polymeric structure (-OH, C-H, C-N, C-O and Cl-C bonds) and the changes observed in the spectra are likely due to the adsorption of components from the effluent as well as chemical reactions due to the presence of potential additives employed during membrane production.

**Figure 6:**
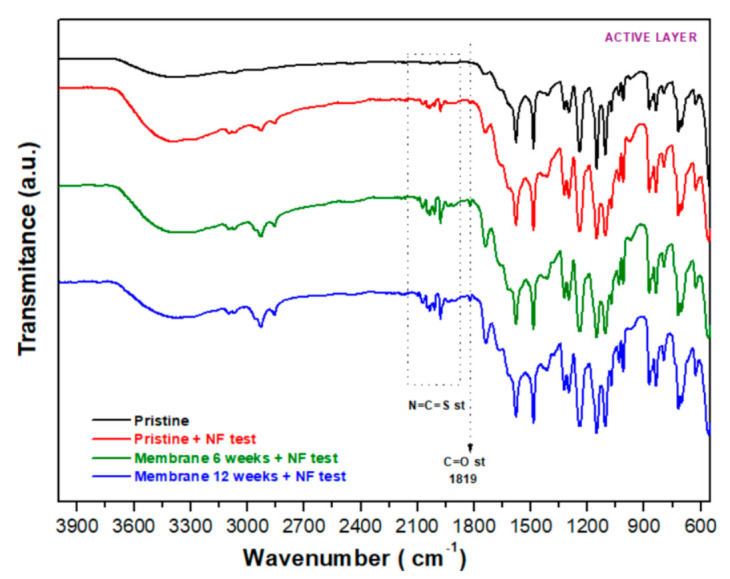
FT−IR spectra of the active layer for the pristine membrane before and after the NF test as well as the active layer of membranes aged for 6 and 12 weeks and after NF tests.

The impact of filtration in the chemical structure of the membrane was also checked by comparing the pristine membrane before and after the NF test as well as two membranes immersed for 6 and 12 weeks prior to an NF test (Figure 6).

Spectra depicted in Figure 6, for all membranes tested by filtration, did not show significant differences apart from the new signals detected in the region of 2090–1952 cm^−1^, which could be assigned to isothiocyanate bonds (N=C=S st) [45,52], and a new peak at 1819 cm^−1^ (C=O st) associated with acid halide or anhydride, which is an indicator of chemical modifications in the urethane structure. The intensity of the peaks at around 3413 cm^−1^, around 2960–2854 cm^−1^ and around 1737 cm^−1^ (Figure 6) increases as the contact time with the effluent increases. Thus, similarly to the analysis shown in Figure 5, the presence of the bands at 1410 cm^−1^ and 1377 cm^−1^ is probably related to the presence of alcohols and effluent components (Figure S4).

**Figure 7:**
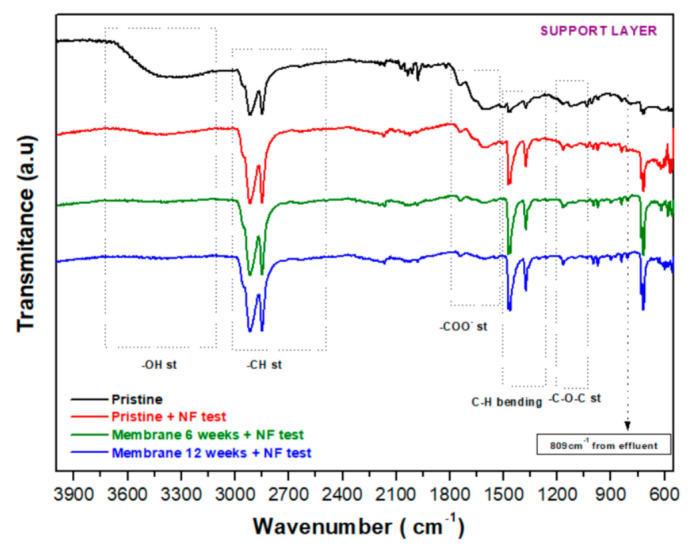
FT−IR spectra of the support layer in the pristine membrane before and after NF test, as well as aged FT−IR spectra of the support layers for membranes aged for 6 and 12 weeks used in NF tests.

The support layers of the membranes were also analyzed and compared by FT−IR (Figure 7). First, the chemical structure for pristine and aged duplicate membranes (named Membrane 1 and Membrane 2), immersed in caustic effluent for 12 weeks (Figure S5), was analyzed. Similarly to the analysis conducted for the active layer (Figure 6), FT−IR characterization was conducted for the support layer in aged membranes immersed for 6 and 12 weeks in caustic effluent and subjected to NF tests (Figure 7), and the results were compared to NF-tested and untested pristine membrane support.

The spectra for the support layer of membranes immersed for 12 weeks in spent caustic effluent were identical to those of the pristine membrane and showed peaks associated with C-H bonds (2985–2800 cm^−1^, 1457 cm^−1^ and 1383 cm^−1^) that remained in the main structure. However, an evident deacetylation with the loss of -OH (region around 3400 cm^−1^) and COO- groups (around 1660 cm^−1^) and an important change in the region for C-O bonds (1165–1015 cm^−1^) groups indicates that the polymeric structure of the support layers might have been considerably damaged after exposure to the spent caustic effluent.

As expected, these changes in the chemical structure were similar to those found in submerged membranes subjected to filtration (Figure 7). The peak at 809 cm^−1^ present in both spectra (Figure S5 and Figure 7) could be also related to the detected effluent components (Figure S4).

As a conclusion, the data discussed above suggest that the active layer is not likely to have been modified since the differences observed are related to the adsorption of organic components that are present in spent caustic, affecting its retentive properties, as shown above. On the other hand, the support layer seems to have been considerably altered, in line with the changes observed in the permeability of the membranes, porosity properties and EDS results.

### 3.3. Inopor Ceramic Membrane

Similarly to what has been shown in Section 3.2, the performance of the ceramic membrane was also evaluated.

Although compaction is not required for ceramic membranes, this membrane was allowed to compact for half an hour, with DI water. Hydraulic permeability was determined to be 5.61 ± 0.14 Lh^−1^bar^−1^m^−2^ at 23 ± 2 °C. The fabricant of the ceramic membranes, Inopor, lists a hydraulic permeability of 9.0 Lh^−1^bar^−1^m^−2^, which is relatively higher than the obtained value, but typically ceramic membranes may present a slight deviation due to the rigid structure [32].

**Figure 8:**
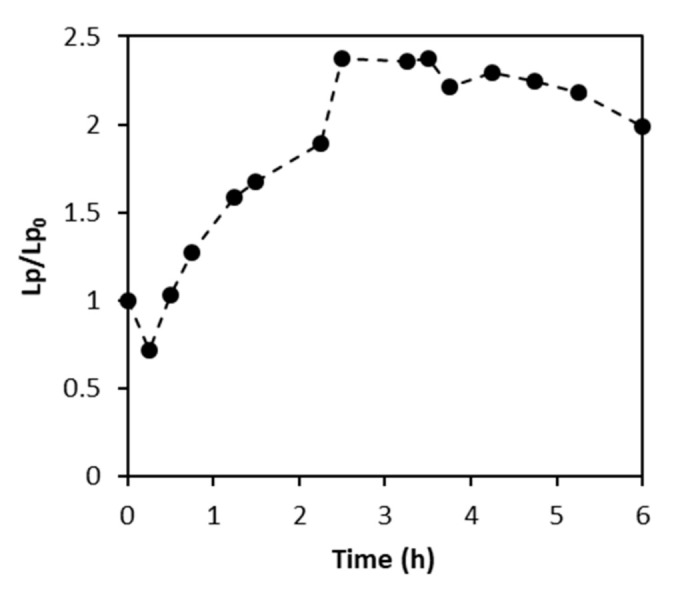
Relative permeability of the 200 Da ceramic membrane throughout the test with spent caustic effluent.

Tests were performed for a maximum period of 6 h. Figure 8 shows the permeability evolution for the ceramic membrane during the NF test.

Figure 8 shows that permeability increased between 0.5 and 2.5 h of testing, slightly decreasing afterward for the 200 Da membrane. TiO_2_ is a nontoxic material, and according to the manufacturer, it is very resistant to extreme pH conditions (1–13 between 20 to 40 °C) [53]. Spent caustic is a difficult wastewater due to extreme pH, around 14 (Table 1). Contrary to expectations, the 200 Da ceramic membrane did not resist spent caustic.

The results obtained evidence that the membranes addressed are not suitable to treat the tested spent caustic effluents. After the filtration test, the membrane was rinsed with DI water and *L_P_* was assessed. For the 200 Da membrane, *L_P_* was determined to be 47.72 ± 1.23 Lh^−1^bar^−1^m^−2^, at 22 ± 2 °C. The original hydraulic permeability was determined as 5.61 ± 0.14 Lh^−1^bar^−1^m^−2^ at 23 ± 2 °C, from where it may be observed that L_P_ increased more than 8 times, not being able to recover the initial permeability.

**Figure 9:**
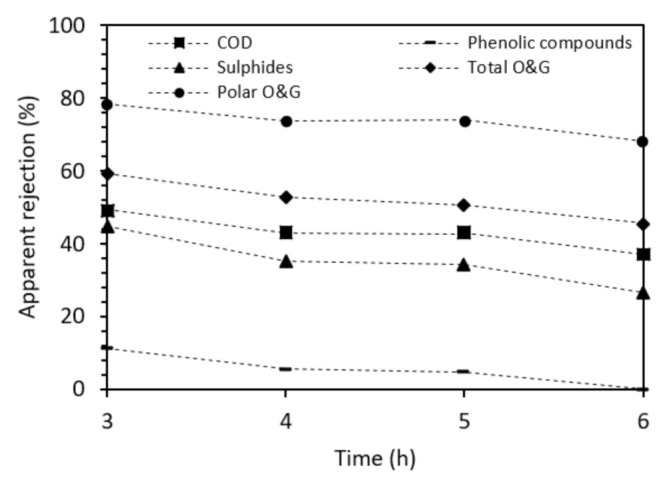
Apparent rejection of contaminants during separation with the 200 Da ceramic membrane.

Figure 9 shows the evolution in the rejection of all contaminants (or lumped parameters) addressed (COD, phenolic compounds, sulfides, total O&G and polar O&G) throughout the last 3 h of the test (because the flux was too low before that to allow for a permeate collection).

The loss in retentive properties could be related to changes in the membrane structure. Considering the rejection capabilities of the ceramic membrane, phenolic compounds seem to be the contaminants with the lowest rejection, possibly due to their low molecular weight. The comparison of the removals obtained for total O&G and polar O&G suggests that the membrane performed better towards the removal of polar O&G. Some authors argue that NF TiO_2_ ceramic membranes possess a tendency to lose rejection when the contaminants are positively charged and maintain high rejection for negatively charged compounds and low molecular weight neutral organics [54]. In a strongly alkaline medium, organic compounds with functional groups located at the end of their structures are usually present in their ionic form.

In order to further study the reasons behind the loss of rejection and to confirm if loss of membrane integrity could have occurred, an ICP analysis was performed on the feed, permeate and retentate samples (Table 4). SEM–EDS analyses were also performed on the membrane.

Table 4 shows a similar aluminum concentration in both the feed and retentate, but a slightly lower aluminum concentration in the permeate samples, therefore showing that there was no release of aluminum from the membrane structure. For the titanium element, concentration was slightly increased in the retentate compared with feed concentration, but concentration increased considerably in the permeate samples, leading to an indication of titanium being released from the membrane structure.

**Figure 10:**
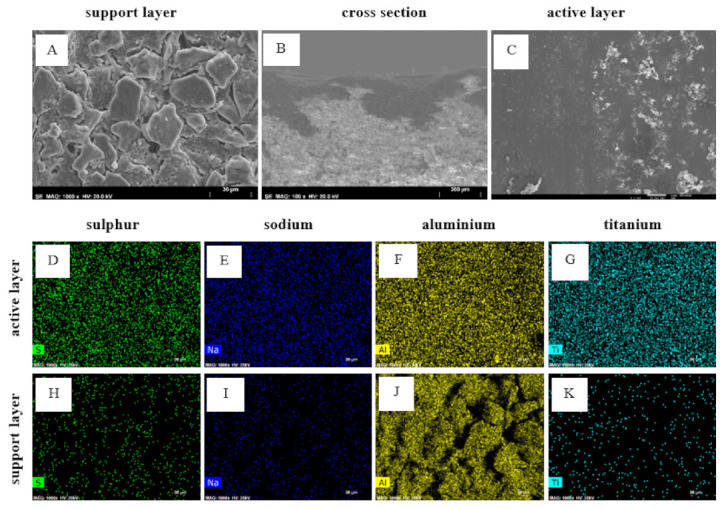
SEM-EDS analysis of the 200 Da ceramic membrane after the NF test as well as the mapping of the most significant elements on the active layer and support layer after NF test. (**A**) Support layer; (**B**) cross-section; (**C**) active layer; (**D**) sulfur element on the active layer; (**E**) sodium element on the active layer; (**F**) aluminum element on the active layer; (**G**) titanium element on the active layer; (**H**) sulfur element on the support layer; (**I**) sodium element on the support layer; (**J**) aluminum element on the support layer; (**K**) titanium element on the support layer. Layer magnifications are ×1000 and cross-section magnification is ×100.

Cross-sections, surfaces and element mapping of the ceramic membrane were analyzed by SEM. Figure 10 shows the surfaces and cross-section of the membrane, along with the mapping of its most significant elements. Table 5 contains the EDS analysis for the active layer of the ceramic membrane after the NF tests.

Although SEM analysis (Figure 10) does not appear to evidence damages on the membrane structure (e.g., cracks) or differences in the distribution of the elements over the membrane, the increase in the permeability of the membrane and the loss of its retention properties is corroborated by ICP observations, which may indicate a disintegration mechanism. Possibly, because the loss of Ti detected by ICP from the membrane to the permeate is not so high and/or can be homogeneous throughout the membrane area, SEM-EDS images do not show it as ICP analysis did. Although the membrane was thoroughly rinsed with DI water after the experiment, the deposition of some salts could be observed in both the active and support layers. The distribution of some elements originally only present in spent caustic, such as sulfur and sodium, was analyzed through mapping. The distribution of the metallic elements that compose the membrane (aluminum and titanium) was also analyzed by mapping. The concentration of sulfur and sodium is considerably higher in the selective layer, which might indicate a higher retention capacity for these compounds. The aluminum distribution seems to be similar on both sides of the membrane. Likewise, titanium is also present in both layers. ICP results indicate a loss of only titanium. Nevertheless, the presence of aluminum occurs in both layers due to a deposition of compounds from the matrix on the active layer, where there is the aluminum element. It is possible that the loss of elements observed by ICP has no such expression in the Figure 10 results, presumably because this loss might be not so relevant. Due to this membrane presenting high rejections, the concentration of elements on the active layer is expected to be higher. On the other hand, the support layer pores are wider and, therefore, may accumulate some elements from the active layer, which could mean that the titanium element released by the active layer will accumulate in the support layer.

## 4. Conclusions

Hydraulic permeability results of the polymeric membrane showed a noteworthy decrease in performance, mainly during 6 weeks of aging, and a smaller loss of permeability in the second period of 6 weeks (final permeability was 4.6 times higher than the hydraulic permeability of the pristine sample). A significant loss in rejection was also observed, following the permeability results (between 5 and 20% less rejection in the analyzed contaminants/lumped parameters). SEM analysis showed that the cross-section of the membrane presented a different pore density, as if the structure loosens up with time in direct contact with spent caustic. Thickness was also decreased during aging. EDS analyses indicated an increase in the carbon element on the active layer, while FT−IR results suggest that the active layer did not present any structural modifications; however, the adsorption of the organic compounds naturally present in spent caustic may have been responsible for the selectivity decay. On the other hand, the support layer presented several modifications that may justify the permeability changes observed.

The hydraulic permeability of the 200 Da ceramic membrane decreased during the first 10 min of testing, but after that, the membrane started increasing in permeability regularly (and lost rejection properties) until 2.5 h of testing, contrary to expectations. EDS and SEM mapping results suggest the disintegration of the membrane, corroborated by the loss of titanium during the NF test. This membrane could be further tested in the future with spent caustic modified to lower pH values, given that the original pH of spent caustic is extremely high.

## Figures and Tables

**Table 1:** Characteristics of spent caustic effluents tested.

Effluent No.	1	2
Date of collection	11 January 2019	10 August 2019
COD (mg O_2_/L)	102,190	88,360
Phenolic compounds (mg/L)	1908	2742
Sulfides (mg/L)	34.9	31.6
Total O&G (mg/L)	18,970	17,837
Polar O&G (mg/L)	15,053	10,090
Nonpolar O&G (mg/L)	3917	7747
Conductivity (mS/cm)	60.7	61.2
pH (Soresen scale)	13.9	13.9

**Table 2:** EDS analysis of active and support layers of polymeric membranes (pristine, 6 and 12 weeks aged prior to NF test).

Membrane	Active Layer			Support Layer	
	C (%)	S (%)	O (%)	C (%)	S (%)
Pristine membrane	61.6	19.9	18.5	99.0	1.0
Aged for 6 weeks	64.7	19.0	16.3	99.0	1.0
Aged for 12 weeks	67.7	16.6	15.7	99.3	0.7

**Table 3:** Pore measurements for the support layer of the pristine membrane, as well as for the 6 and 12 weeks aged membranes prior to NF test based on the SEM cross-section ×300 magnification.

	Pristine	Membrane 6	Membrane 12
Porosity (%)	12.10	19.91	27.93
Number of pores	2196	2962	4039
Pore density (µm^−2^)	0.032	0.041	0.063
Minimum pore area (µm^2^)	0.074	0.074	0.075
Maximum pore area (µm^2^)	442.45	3821.37	714.92
Total pore area (µm^2^)	8345.47	14,257.44	18,022.10
Average circularity	0.828 ± 0.252	0.878 ± 0.221	0.837 ± 0.252
Minimum circularity	0.078	0.038	0.007
Maximum circularity	1.000	1.000	1.000
Average Feret diameter (µm)	1.662 ± 3.745	1.441 ± 4.974	1.503 ± 3.570
Minimum Feret’s diameter (µm)	0.384	0.384	0.386
Maximum Feret’s diameter (µm)	39.761	141.438	66.347

**Table 4:** ICP analysis of feed, retentate and permeate samples after/during the NF test with the 200 Da membrane.

Sample Name	[Al], mg/L	[Ti], mg/L
Feed (spent caustic)	2.06	0.02
Retentate—Total *	2.07	0.05
Permeate—Total *	1.38	0.43
Permeate at 3 h of NF test	1.45	0.50
Permeate at 4 h of NF test	1.37	0.29
Permeate at 5 h of NF test	1.62	0.27
Permeate at 6 h of NF test	1.81	0.28

* The analyses refer to the total volume of retentate/permeate collected during the 6 h of the NF test.

**Table 5:** EDS analysis of active layer of the ceramic membrane after the NF test (200 Da).

Membrane	O (%)	C (%)	Al (%)	Ti (%)	Zr (%)	Na (%)	S (%)	Fe (%)	Si (%)
200 Da	22.1	10.2	18.0	23.8	10.8	9.9	3.2	1.6	0.4

## Supplementary Materials

The following supporting information can be downloaded at: https://www.mdpi.com/article/10.3390/membranes12010098/s1, Figure S1: SEM binarized cross section images, using a threshold of 149 for Pristine Membrane (up), and 255 for Membrane 6 (middle) and Membrane 12 (down); Figure S2: Representation of the circularity (left) and porous area (µm^2^) (right) distribution, for all porous (counts) detected by the ImageJ software: Pristine (Up), Membrane 6 (middle) and Membrane 12 (down). (Obtained with Origin Software); Figure S3: FT-IR spectra magnifications in regions 4000–2700 cm^−1^ (A) and 2200–550 cm^−1^ (B) of the active layer of the pristine membrane and membranes after 12 weeks immersion in spent caustic (made in duplicate); Figure S4: FT-IR spectra of spent caustic samples collected in the 17 March 2018 in original form (A) and acidified form (C) and collected in the 22 March 2018 in original form (B) and acidified form (D); Figure S5: FT-IR spectra of support layer for pristine membrane and membranes (made in duplicate) after 12 weeks immersed in spent caustic effluent.

## Nomenclature

*A*	Filtration area (m^2^)
*L_P_*	Hydraulic permeability (Lh^−1^bar^−1^m^−2^)
*L*_*P*0_	Hydraulic permeability for the pristine membrane (Lh^−1^bar^−1^m^−2^)
TMP	Transmembrane pressure (bar)
Δ*t*	Duration of nanofiltration test (h)
*V*	Permeate volume (L)
*Acronym list*	
ATR	Attenuated total reflectance
COD	Chemical oxygen demand
DI	Distilled
EDS	Energy dispersive spectroscopy
FT−IR	Fourier-transform infrared spectroscopy
ICP-AES	Inductively coupled plasma atomic emission spectroscopy
MWCO	Molecular weight cut-off
NF	Nanofiltration
O&G	Oil and grease
PAHs	Polycyclic aromatic hydrocarbons
SEM	Scanning electron spectroscopy
SDD	Silicon drift detector
SMEWW	Standard method for examination of water and wastewater
PES	Polyethersulfone
PS	Polysulfone
