# Supplementary material for: Assessment of the Potential of Using Nanofiltration Polymeric and Ceramic Membranes to Treat Refinery Spent Caustic Effluents
Source: Membranes (Basel). 2022 Jan 17;12(1):98. doi: 10.3390/membranes12010098 (PMC8779506; doi:10.3390/membranes12010098)
Supplement: Supplementary file 1 [file membranes-12-00098-s001.zip › membranes-1512234-supplementary.pdf]

Supplementary Material

Assessment of the Potential of Using Nanofiltration Polymeric and Ceramic Membranes to Treat Refinery Spent Caustic Effluents

Ana Isabel Rita [1,2,3], Ana Rita Nabais [3], Luisa A. Neves [3], Rosa Huertas [3,4], Maria Santos [1], Luis M. Madeira [2] and Sandra Sanches [4,*]

[1] Sines Refinery, Petrogal S.A., 7520-952 Sines, Portugal; aibr1910@gmail.com (R.A.I.); maria.santos@galp.com (S.M.)

[2] LEPABE-Laboratory for Process Engineering, Environment, Biotechnology and Energy, Faculty of Engineering, University of Porto, Rua Dr. Roberto Frias, 4200-465 Porto, Portugal; mmadeira@fe.up.pt

[3] LAQV/REQUIMTE, Chemistry Department, Nova School of Science and Technology, Universidade NOVA de Lisboa, 2829-516 Caparica, Portugal; a.nabais@campus.fct.unl.pt (N.A.R.); lan11892@fct.unl.pt (N.L.A.); rosa.huertas@ibet.pt (H.R.M.)

[4] IBET-Instituto de Biologia Experimental e Tecnológica, Apartado 12, 2780-157 Oeiras, Portugal

* Correspondence: sandramsanches@gmail.com

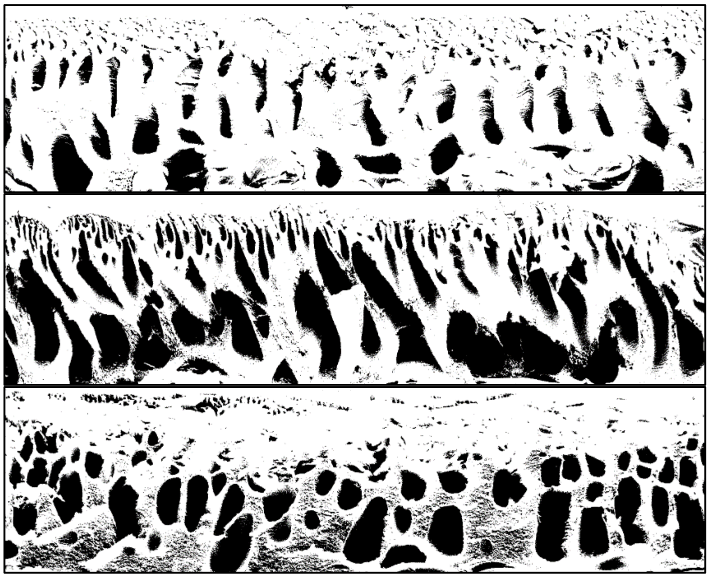

Figure S1. - SEM binarized cross section images, using a threshold of 149 for Pristine Membrane (up), and 255 for Membrane 6 (middle) and Membrane 12 (down).

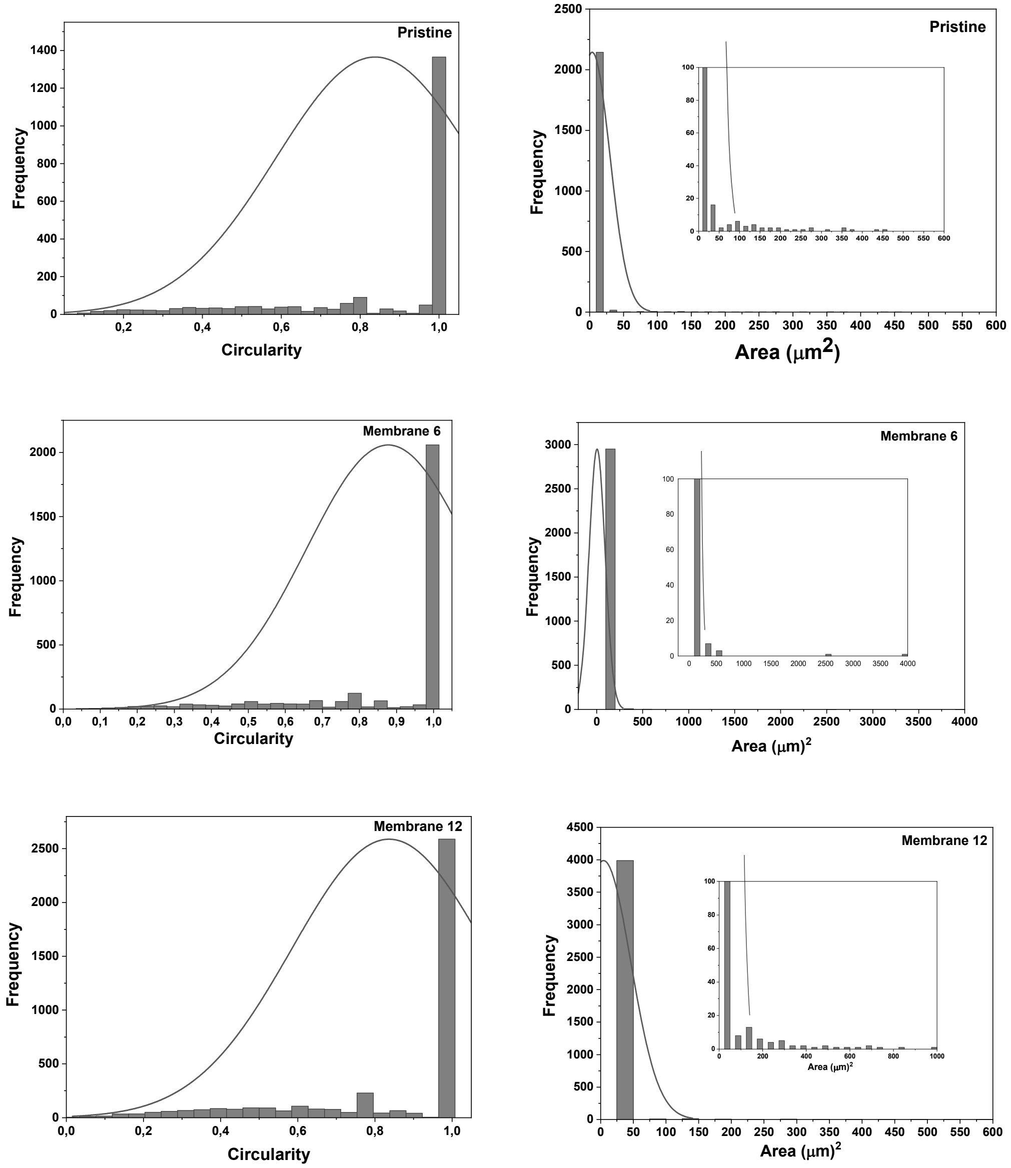

Figure S2. - Representation of the circularity (left) and porous area (μm2) (right) distribution, for all porous (counts) detected by the ImageJ software: Pristine (Up), Membrane 6 (middle) and Membrane 12 (down). (Obtained with Origin Software).

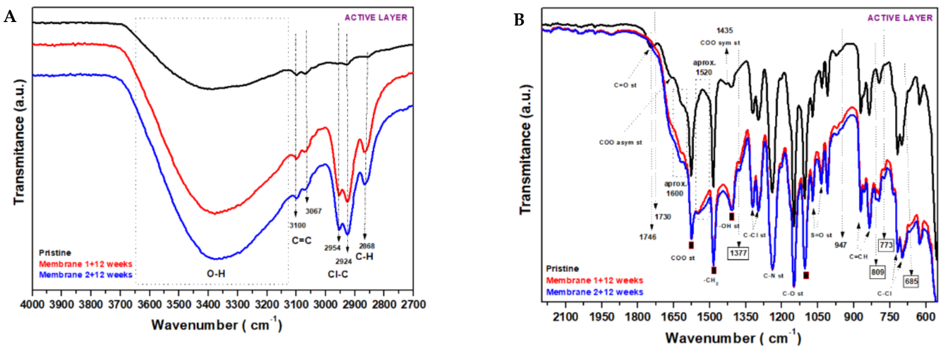

Figure S3. - FT-IR spectra magnifications in regions 4000–2700 cm-1 (A) and 2200–550 cm-1 (B) of the active layer of the pristine membrane and membranes after 12 weeks immersion in spent caustic (made in duplicate).

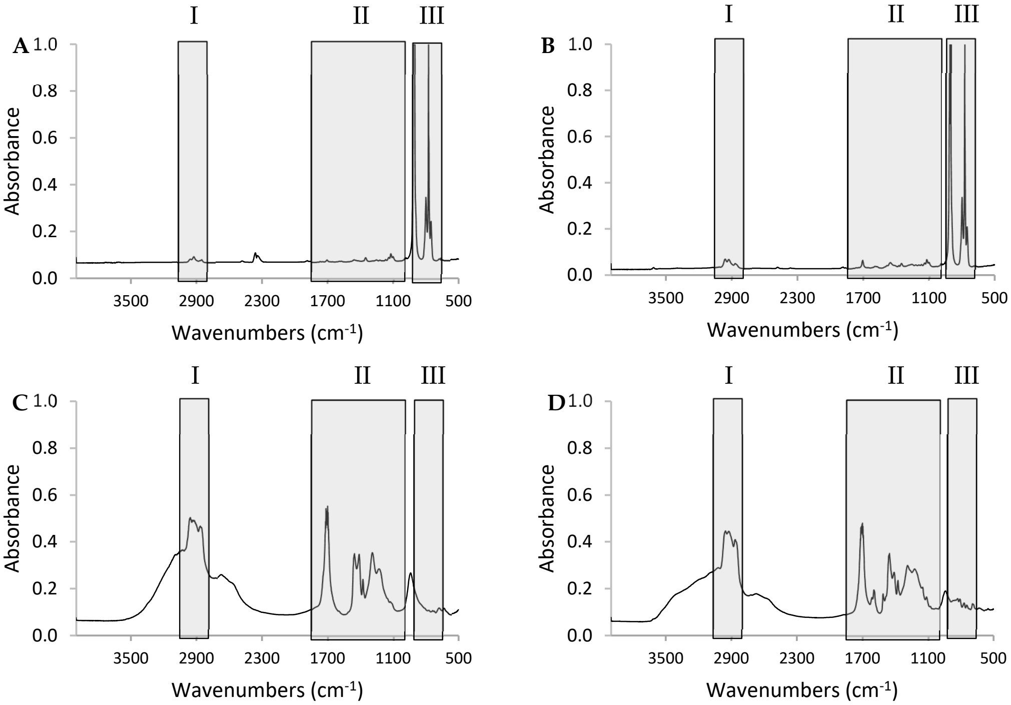

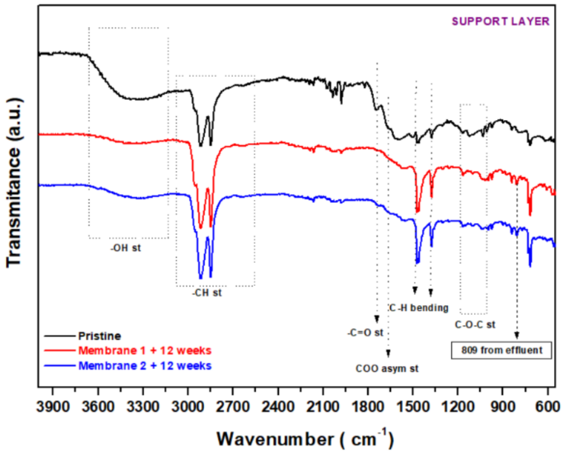

Figure S5. - FT-IR spectra of support layer for pristine membrane and membranes (made in duplicate) after 12 weeks immersed in spent caustic effluent.
